# Neurotrophins Role in Depression Neurobiology: A Review of Basic and Clinical Evidence

**DOI:** 10.2174/157015911798376262

**Published:** 2011-12

**Authors:** Fani L Neto, Gisela Borges, Sonia Torres-Sanchez, Juan A Mico, Esther Berrocoso

**Affiliations:** a Instituto de Histologia e Embriologia, Faculdade de Medicina e IBMC, Universidade do Porto, 4200-319, Porto, Portugal; b Department of Neuroscience (Pharmacology and Psychiatry), Faculty of Medicine, University of Cadiz, 11003 Cadiz, Spain; c Ciber of Mental Health (CIBERSAM), Instituto de Salud Carlos III, Madrid, Spain

**Keywords:** Antidepressants, BDNF, depression, hippocampal neurogenesis, neuropsychiatric disorders, stress, Val66Met polymorphism, VGF.

## Abstract

Depression is a neuropsychiatric disorder affecting a huge percentage of the active population especially in developed countries. Research has devoted much of its attention to this problematic and many drugs have been developed and are currently prescribed to treat this pathology. Yet, many patients are refractory to the available therapeutic drugs, which mainly act by increasing the levels of the monoamines serotonin and noradrenaline in the synaptic cleft. Even in the cases antidepressants are effective, it is usually observed a delay of a few weeks between the onset of treatment and remission of the clinical symptoms. Additionally, many of these patients who show remission with antidepressant therapy present a relapse of depression upon treatment cessation. Thus research has focused on other possible molecular targets, besides monoamines, underlying depression. Both basic and clinical evidence indicates that depression is associated with several structural and neurochemical changes where the levels of neurotrophins, particularly of brain-derived neurotrophic factor (BDNF), are altered. Antidepressants, as well as other therapeutic strategies, seem to restore these levels. Neuronal atrophy, mostly detected in limbic structures that regulate mood and cognition, like the hippocampus, is observed in depressed patients and in animal behavioural paradigms for depression. Moreover, chronic antidepressant treatment enhances adult hippocampal neurogenesis, supporting the notion that this event underlies antidepressants effects. Here we review some of the preclinical and clinical studies, aimed at disclosing the role of neurotrophins in the pathophysiological mechanisms of depression and the mode of action of antidepressants, which favour the neurotrophic/neurogenic hypothesis.

## HYPOTHESIS OF DEPRESSION: A GENERAL OVERVIEW

Depression is an illness affecting a great number of people worldwide. This problematic involves not only the individuals, but also their families and the surrounding social environment. Taking as a reference the United States of America, it is proposed that about one in six individuals will deal with clinically diagnosed symptoms of depression during their lifetime [1]. Apart from the disheartened mood, the loss of interest or pleasure, feelings of guilt or worthlessness, disturbed sleep or appetite, low energy, poor concentration and suicidal intentions are some of the principal symptoms observed in depressed patients [2, 3]. This unpleasant “state of mind” may be related with working conditions, self-perceived stress, anxiety and quality of life [4], however, it’s curious and intriguing why predisposition to develop depression is higher in certain persons than others. Levinson [5] proposed that a great part of depression is genetically determined. In this meta-analysis there are positive associations with some psychiatric disorders (bipolar disorder, suicidal behaviour, and depression-related personality traits) and a polymorphism in the serotonin transporter promoter region (5-HTTLPR). Nowadays, it’s assumed that a complex interaction between genetic, biochemical and environmental factors may be underlying the causative aetiology of this disorder [3].

The study of depression keeps being a challenge for those who want to reveal the real mechanism of this psychiatric disorder. Indeed, in spite of the long history of research in this field, the science behind mood regulation remains practically unknown and the development of efficient drugs is far from being satisfactory [6-8]. One of the reasons appointed to this lack of knowledge is the difficulty associated with the observation of pathological changes within the human brain [9]. At the moment, studies on humans rely on the evaluation of cases in which certain brain structures are absent (accidently or not) or on post-mortem tissues which, by law, are only available for experimental tests several hours after death. In this matter, the development of animal models of depression has helped to attain some important achievements. However, to be considered a valid animal model of depression it needs to satisfy some requirements, initially proposed by McKinney and Bunny [10, 11]. Its undeniable the value of animal models of depression in the identification and validation of monoamine-based antidepressant compounds, although inherent limitations of animals models in comparison to the manifestations of such a complex disease in humans must be accepted [10]. Despite the difficulty of researching in this area, scientists have been improving their knowledge and acquiring new methods and approaches, testing new concepts and drugs that certainly will be useful in the future.

The monoamine hypothesis of depression was the most accepted by the scientific community [12-14] which defended that this illness was caused by a deficit in the neurotransmission of serotonin and noradrenaline and that it could be reversed by drugs (namely antidepressants) that promote the increase of these molecules in the synaptic cleft [9, 15, 16]. However, this theory was not sufficient to explain all the mechanisms beyond depression. Antidepressants acting either by blockage of the reuptake of monoamines or by inhibition of their degradation at the synaptic cleft promoted an immediate increase of serotonin and noradrenaline transmission [9, 15, 16] but the antidepressant effect was only saw after a few weeks of treatment. Monoamine depletion studies demonstrate decreased mood in subjects with a family history of major depression (MD) and in drug-free patients with MD in remission, but do not decrease mood in healthy humans [17, 18]. So, it soon became obvious that depression involves further modifications besides those at the monoamine system. At the same time several studies emerged to assess new pharmacological models that may help to understand the mechanisms and pathophysiological changes leading to a depressive behaviour. 

## THE NEUROTROPHIC HYPOTHESIS OF DEPRESSION

Several studies have led to the formulation of the *Neurotrophic Hypothesis of Depression*, which postulates that low levels of brain-derived neurotrophic factor (BDNF) lead to a depression condition [19, 20]. Neurotrophins are growth factors with crucial roles in the formation and plasticity of neuronal networks [21]. The neurotrophin family include nerve growth factor (NGF), BDNF, neurotrophin-3 (NT-3) and neurotrophin-4 (NT-4). They are initially synthesized as precursor proteins (proneurotrophins), which are processed extracellular or intracellularly to be secreted mostly in a mature, biologically active form [22-26]. The neurotrophins show binding specificity for particular tyrosine kinase receptors (Trk), and non-specificity for the p75 neurotrophin receptor (p75NTR) (see for review [27]). In particular, pro-BDNF preferentially activates p75NTR receptor to mediate programmed neuronal death [28], to decrease dendrite complexity and spine density in hippocampal neurons [29], and to induce long-term depression of synaptic transmission [30, 31]. On the contrary, mature BDNF (mBDNF) selectively activates TrkB, a member of the tyrosine kinase receptors family, to promote survival and differentiation, increasing the branching of axons and dendrites and stabilizing synaptic contacts [32]. Thus, due to the essential role of BDNF for cell differentiation, nerve growth and neuronal survival it has been implicated in several brain diseases, including depression. 

In what concerns to stress, a prominent cause of depression, it is well established its relation with low levels of BDNF expression. For example, stress caused by immobilization decreases the expression of this neurotrophin in the hippocampus [33]. Nowadays, this vision has been modified, highlighting the role of BDNF (the most studied neurotrophin) in the adaptation of neural networks that are responsible for different aspects of mood regulation [19, 34, 35]. In the last few years, solid evidences have been suggesting an important role of neurotrophins in mood regulation and antidepressant-like behaviour [36, 37]. Indeed, depressed patients have decreased serum levels of BDNF [38] while the treatment with antidepressants promotes an increase in these levels [38]. However, these observations are not totally accepted as a proof of BDNF involvement since neurotrophins are mainly involved in the neural development, function and plasticity [39-45] which are some of the points of action of the treatment with antidepressants [19, 46]. Several authors have reported structural and functional BDNF-induced modifications of neuronal networks, which allowed their re-organization as a response to inputs from the environment [43, 47, 48]. These effects were produced by binding and activation of tyrosine kinase receptor subtype B (TrkB) leading to changes in cell shape and gene transcription [47]. Antidepressants were also shown to be able to interfere with neuronal plasticity [19, 46] being the neurogenesis the most interesting phenomenon within this field [49]. So far, neuroscientists have been proposing new mechanistic hypothesis for depression, in search for clues for understanding its pathophysiology. A different point of view actually discusses the balance between neuronal death and neurogenesis as an etiological cause of this pathology [50, 51]. In fact, neurogenesis is a possible phenomenon in adults [52-54] and neurotrophins are important for nervous cells formation and differentiation [55, 56]. Several studies have been proposed to test some of these theories relating the balance between neuronal death and neurogenesis, but most of them still lack experimental verification [57, 58]. In rodent hippocampus, for example, it has been observed that neurogenesis is increased by antidepressant treatments [46, 59-61]. Additionally, several studies correlate antidepressants administration with neurotrophins expression. Interestingly, BDNF expression was dependent on a chronic treatment [62] which was very curious given the time that antidepressants need to have a therapeutic effect. In fact it is possible that, somehow, neurotrophins may also have functions in the regulation of transmitter release. Actually, several studies have reported this role. After Lohof and colleagues [63] demonstrated a presynaptic mechanism of enhancement of synaptic transmission promoted by BDNF and NT-3, other authors reached similar conclusions [64]. Later it was found that dysregulation of presynaptic TrkB (truncated form) in the hippocampal neurons reduced the amount of BDNF-induced neurotransmitter release [65, 66] and that the treat-ment of cultured hippocampal neurons with BDNF promoted a decrease in paired pulse facilitation [67-68]. Tyler and colleagues proposed that BDNF alters the mode of vesicle retrieval from a slow complete fusion with membrane [69] to a faster vesicle retrieval without complete fusion [70] modulated by presynaptic voltage-dependent Ca^2+^ channels. These effects appear to be related with TrkB-dependent phosphorylation [71-73]. Consequently, the number of docking sites increases promoting neurotransmitters vesicle’s mobilization to the active zone. In summary it appears that neurotrophins, and in particular BDNF are engaged in the pathophysiology of several neurologic diseases, including depression, by acting through several mechanistic approaches implicated in neuronal death, neurogenesis, control of neurotransmitters release or even the induction of certain neuropeptides, as VGF (non-acronym). Indeed, the finding of increases in the expression of BDNF and of its principal receptor, TrkB, after antidepressant treatment marked the beginning of this line of research [38, 62, 74] and of the neurotrophic hypothesis of depression.

## BDNF IN DEPRESSION: PRECLINICAL AND CLINICAL EVIDENCES

### Preclinical Evidences

#### Regulation of BDNF in Animal Models of Stress

It is widely known that stress is a risk factor for major depression in individuals with genetic vulnerability. Basic research has used animal models which imply stress to model such complex multi-syndrome psychiatric illnesses like major depression because they induce or mimic some of their clinical symptoms: anhedonia, sleep disturbances, increased corticosterone levels, etc. Thus, numerous studies have focused in the role of BDNF in different animal models of stress and antidepressant drugs response. In general, most of the studies have shown that acute or chronic stress induced by different types of stressors, such as immobilization, unpredictable, footshock, social isolation, social defeat, maternal deprivation, restraint and swim stress decrease BDNF levels in the hippocampus (see Table **1** for references). In fact, significant reductions of BDNF messenger RNA (mRNA) were observed in CA1 and CA3 pyramidal cell layers but the greatest effects were found in the dentate gyrus granule cell layer [75]. Given that in this last layer is where adult neurogenesis process occurs, it is suggested a relationship between BDNF and neurogenesis (see below in the section Hippocampal Neurogenesis). Thus, as cited above, BDNF appears to mediate neurogenesis in dentate gyrus and neuronal atrophy seen with stressful conditions seems to occur in CA3 pyramidal cells. Indeed, stress reduces the length and number of branch points of CA3 neurons in the hippocampus [76]. Atrophy or remodelling of CA3 neurons could contribute to the reduction in hippocampal volume reported in depressed patients [77, 78]. However, there is no common consensus in the literature about the decrease of BDNF expression in stress-related animal models. For example, chronic restraint stress for 21 days did not modify BDNF expression in Kuroda and McEwen’s studies [79] (Table **1**). In contrast, a few years later, Murakami and co-workers [80], applied the same stress paradigm and a significant decrease of BDNF expression in the hippocampus was found. Such discrepancy also occurs in several studies that also reported no effects or even increase of BDNF levels in the hippocampus following exposure to chronic stress [81-87] (Table **1**). The authors argue that the differences found reside in the stress protocols applied in each study. In conclusion, the effects of stress on BDNF mRNA expression in the hippocampus appear to be dependent on several factors: (i) the type of stressor; its intensity, duration, frequency, number of exposures, etc. [88, 89]; (ii) the procedure used to quantify BDNF levels and (iii) the isoform or isoforms measured in each assay [25, 90]. In addition to physical stress models, there are also available data about corticosterone levels modulation and BDNF. It is widely known that stress increases plasma and adrenal corticosterone levels [80, 91] and several studies reported an inverse correlation between stress/corticosterone levels and BDNF expression in the hippocampus. Thus, corticosterone administration (which mimics stress situation) decreases BDNF levels in the hippocampus [33, 80, 92-95]. In contrast, in adrenalectomized animals, hippocampal BDNF values were restored to baseline levels [33, 95]. However, stress in adrenalectomized rats decreases BDNF expression in the dentate gyrus, where main neurogenesis process occurs, but not in CA1 and CA3 layers of the hippocampus [33]. Thus, in summary, although with some significant disagreements, it seems that there is a correlation between stress induction and BDNF decreased expression mainly in the dentate gyrus as it is shown in Table **1** and in less extension in CA3 and CA1 layers. 

#### Stress and BDNF, Possible Mechanisms

What are the possible mechanisms underlying the putative down-regulation of BDNF expression due to stress exposure? As we mentioned before several studies have demonstrated that stress causes impaired neurogenesis and atrophy in certain limbic structures, particularly the hippocampus, and that BDNF down-regulation is one of the events that occurs. However, the precise mechanism underlying this down-regulation has not been fully understood. The studies available suggest that stress induces lasting down-regulation of BDNF transcripts III and IV and robust chromatin modification [96, 97]. So, stressful experience would decrease levels of specific BDNF isoforms and could contribute to the atrophy of limbic structures, including that in the hippocampus that has been observed in depressed patients. However, this BDNF regulation system seems to be very complex because several factors influence it. Thus, it has been suggested that glucocorticoids modulate BDNF signalling pathways. It has already been mentioned that high adrenal-glucocorticoid levels, which is one of the hallmark endocrine responses to stress, decreases BDNF expression. The mechanisms underlying these phenomena needs to be studied further, but it has been suggested that glucocorticoid inhibits the BDNF-dependent up-regulation of synaptic proteins *via *suppressing the activation of Mitogen-Activated Protein Kinases/Extracellular signal-Regulated Kinases (MAPK/ERK) pathway [98]. Another line of evidence supporting a role of BDNF in depression is the trophic effect of BDNF on the serotoninergic system. For example, the antagonism of serotonin 2A receptors (5-HT_2A_) by ketanserin partially blocks the effect of stress on BDNF expression [99, 100], suggesting that serotonin is a key mediator, an idea congruent with the numerous findings about both serotonin and the 5-HT_2A_ receptor in the aetiology and treatment of depression and other affective disorders [101, 102]. Cytokines have equally recently received much attention in depression field also in relation with BDNF. Studies reveal that the interleukin-1β (IL-1β), cytokine, which is implicated in the development of depressive-like behaviour [103], contributes to BDNF down-regulation. Moreover, the blockade of IL-1β receptors prevented the stress-induced decrease of BDNF [104, 105]. This effect on BDNF transcription could be directly or indirectly mediated by the inhibition of the phosphorylation of cAMP-related element binding protein (CREB, a major transcription factor managing the gene expression of the plasticity-related molecules) [106] or by increasing serotonin extracellular levels in the hippocampus [107], respectively. Together, it suggests that additional components of the stress response may also contribute to the observed changes in BDNF. 

As stated before, most of the studies until now have focused on the effect of BDNF expression level in the hippocampus. However, BDNF is modulated under stress conditions in other brain areas, such as prefrontal cortex. Thus, it has been shown that stress by immobilization produces a significant increase in BDNF expression in prefrontal cortex and the metabotropic glutamate 2/3 receptor (mGluR2/3) agonist, LY35740, attenuates the immobilization stress-induced increase in BDNF mRNA expression in the prefrontal cortex [108]. Therefore, we conclude that stress modulates BDNF expression in the hippocampus and that modulation is affected by several intermediate factors and produced by different signalling pathways. In addition, BDNF expression in other areas apart from hippocampus may play an important role in mood that may be taken in consideration.

#### Antidepressant-Like Effects of BDNF

Following the idea that stressful situations down-regulate BDNF, the effect of BDNF infusion on depressive-like behavioural tests was evaluated. Thus, in 1996, Suiciak and colleagues [109] showed that infusion of BDNF either intracerebroventricularly (i.c.v.) or directly into the rat midbrain (periaqueductal gray matter and dorsal and median raphe nuclei) produced analgesia and, interestingly, increased the activity of the monoaminergic systems. Indeed, BDNF infusion promotes the function, sprouting and growth of serotonin-containing neurons in the brain of adult rats [110] and increases noradrenaline levels in several brain areas including the hippocampus [109]. These effects of BDNF on serotoninergic and noradrenergic system link the classical monoaminergic hypothesis of depression with the neurotrophic theory. Two later studies demonstrated that both acute or subchronic (3-7 days) BDNF infusion into the hippocampus (dentate gyrus and CA3 layer) or in the midbrain, produces an antidepressant-like effect in two behavioural models of depression, the learned helplessness and the forced swimming test paradigms. In these tests, a rodent is subjected to an inescapable stress leading to a “behavioural despair” and, when repeated, the depressive behaviour is increased. Thus, in the learned helplessness test, infusion of BDNF led to a significant improvement of the conditioned avoidance behaviour measured as a decrease in the number of failures and latency to escape, being this effect of a similar magnitude to that of imipramine or fluoxetine effect [37]. In the forced swimming test, acute BDNF infusion also improves antidepressant-like behaviour, decreasing immobility and increasing the swimming behaviour [37, 111]. These two studies suggest a clear antidepressant-like effect of BDNF treatment at midbrain and hippocampus regions, respectively. However, this effect seems to be specific to certain brain areas because BDNF infusion into the ventral tegmental area or in the nucleus accumbens, for example, increases depression like-behaviour. In addition, this behaviour is reversed by the inhibition of BDNF signalling producing an antidepressant-like effect [112]. These findings are in agreement with the stress-induced increase of BDNF expression in prefrontal cortex as mentioned before [108] and are the opposite to the effect observed in the hippocampus, suggesting that BDNF antidepressant effect is area-dependent. On other hand, they could suggest that BDNF downstream signalling mechanisms are differently regulated and that this is the reason for the finding of discordant effects, but it is clear that there is something more to the solely and general enhancement of BDNF levels. 

What seems consistent through the literature available is that BDNF infusion in the hippocampus or/and the midbrain produces antidepressant-like behavioural effects and that most of clinically effective antidepressant drugs work through this mechanism. Thus, considerable work has been devoted to study the effect of antidepressants on BDNF expression, and it appears that antidepressants, even mechanistically different, produce an increase of BDNF mRNA levels in the hippocampus after several weeks of treatment that coincides with the onset of the antidepressant-like effect. 

#### The Relationship Between BDNF and Antidepressants

BDNF modulation could represent a key step in the adaptative changes brought by antidepressants. Indeed, BDNF increase has been reported for selective serotonin reuptake inhibitors (SSRIs), noradrenaline reuptake inhibitors (NRIs), serotonin and noradrenaline reuptake inhibitors (SNRIs) (dual-action antidepressants and tricyclic antidepressants), with monoamine oxidase inhibitors (MAOIs), atypical antidepressants, as well as with electroconvulsive shock treatment, one of the most clinically effective treatments for refractory depression (see Table **1** for references). In addition, other compounds which at some point have been suggested to pose alternative antidepressant mechanisms of action (different to the classical enhancement of monoaminergic system) enhance BDNF expression too (see Table **1**). This is the case of the anaesthetic ketamine and even lithium. Ketamine, a non-competitive antagonist of ionotropic glutamate N-methyl-D-aspartate receptor (NMDA), blocks NMDA receptor signalling leading to an antidepressant effect in both clinical and preclinical studies [113-116]. Lithium has antidepressant efficacy too and glutamatergic NMDA receptor signalling could be the target of its action [117-121]. Thus, different studies have shown that treatment with ketamine or lithium induces an antidepressant-like effect in the forced swimming test paradigm, increasing BDNF protein levels in the rat hippocampus [122-124] (see Table **1**). However, this needs to be further evaluated since recent studies have not found such modification in BDNF levels [91, 114].

Antidepressants, in addition to improve or just modify signalling pathways, may help critical systems to overcome challenging conditions, that is, they may contribute to the neuronal plasticity that is required to cope and adapt to stressful situations. These could be the prime effects on BDNF because it responds to rapid or more prolonged manipulations and represents a key mediator for neuronal plasticity that is necessary to respond to external stimuli like stressful conditions. Thus, how antidepressants modify BDNF levels? Several studies have been performed trying to understand the signalling pathways implicated. The effects on BDNF are complex and may occur at several levels. Therefore, antidepressants appear to increase the tyrosine autophosphorylation of TrkB receptors, activate phospholipase-Cγ (PLCγ) signalling and subsequent phosphorylation of CREB [19]. These data account for the effect of antidepressants in “resting conditions” promoting TrkB activation. However, and more interestingly, are there effects in stress/depression models? Indeed, it has been recently demonstrated that duloxetine chronic treatment specifically increases the mRNA levels of BDNF exon VI and exon IX in the hippocampus upon acute stress, whereas other isoforms like IV were up-regulated both in vehicle and duloxetine-treated animals [96]. These data may account once again for changes in specific BDNF isoforms that may contribute to an adequate response to stress. In conclusion, antidepressants may have a potential impact on activity-dependent plasticity within regions involved in emotional processing, which is compromised in depression. Antidepressants may promote neuroprotective pathways and render them more responsive to preserve cell functionality and overcome cellular resiliency. The ultimate consequence of this is that antidepressants would help the patient to deal with adverse situations.

#### Hippocampal Neurogenesis, Antidepressants and BDNF

Adult neurogenesis is defined as the development of new functioning neurons in the adult brain, which occurs in two neurogenic regions, in the dentate gyrus of the hippocampus and the olfactory bulb. This process has been described for all mammalian species investigated, including non-human primates and humans [125], and although it decreases with age, appears to remain present at low levels even at the oldest ages [125-132]. From these two areas that keep the ability to generate new neurons, the whole attention in depression-related research has been mainly devoted to the hippocampus. 

There are several reasons for this that will be detailed below, but perhaps the surprising up-regulation of neurogenesis by antidepressant treatment, timely coinciding with the onset of the behavioural antidepressant effect, is the most relevant. Thus, we will focus in adult hippocampal neurogenesis in this review. It is originated from precursor cells in the subgranular zone (SGZ) in the dentate gyrus and these new neurons extend dendrites to the molecular layer and axons to the CA3 pyramidal layer, getting morphological and physiological characteristics of adult granule cells. This new neurons in the adult hippocampus might contribute to optimize the strength of the highly plastic mossy fibbers connection between the dentate gyrus and area CA3, and are thus exerting a function at a bottleneck in the hippocampal network. They have been linked to the processing of information in the sense of forming temporal associations and integrating information into contexts, and also play a role in affective behaviour, underscoring the link between cognition and emotion in the hippocampus. Thus, negative emotional interventions, such as stress or depression animal models, have shown to impair neurogenesis as well as BDNF expression in the hippocampus. Indeed, exposure to acute or repeated stress, including social defeat, predator odour, restraint, immobilization, footshock, prenatal stress and chronic mild stress decrease neurogenesis in the adult hippocampus [57]. In addition, Malberg and Duman, have observed that the down-regulation of neurogenesis induced by inescapable stress correlates with behavioural despair in the learned helplessness paradigm [133]. Thus, this established correlation may indicate that there is a relationship between reduced neurogenesis and the behavioural state of the animal. 

As mentioned previously, neurotrophic factors, particularly BDNF [134], are known to be implicated in adult neurogenesis and plasticity. However, evidences suggested that BDNF has a role in neuronal survival but not in proliferation [135], which was later corroborated in BDNF heterozygous and TrkB transgenic mice [136]. Additionally, it is known that neurogenesis is modified by many factors, including aging which dramatically decreases adult hippocampal neurogenesis, and corticoids. In fact, corticoids have been shown to decrease BDNF levels in the hippocampus. In addition, already in 1992, it has been demonstrated that glucocorticoids hormones suppress cell division in the adult dentate gyrus, which indicated that modifying corticosteroids levels might possibly increase hippocampal neurogenesis [137]. It is also recognized that the generation of neurons is increased by environmental and behavioural interventions that have a beneficial effect on mood. Thus, neurogenesis is up-regulated in mice exposed to an enriched environment, in line with an increase in BDNF expression, presenting more new neurons in the dentate gyrus and a larger hippocampal granule cells layer compared with mice housed in standard cages [138, 139]. Physical exercise appears to also have a positive effect on adult hippocampal neurogenesis and BDNF expression as it has been reported that running doubled the number of surviving newborn cell, in amounts similar to enrichment conditions [140, 141]. These preclinical studies go along with clinical evidences showing a therapeutic response of exercise on mood in depressed patients [142-147]. Altogether, it appears that newly generated neurons in the dentate gyrus in the adult hippocampus are affected by the hippocampal-dependent memory formation and BDNF regulation and may also be implicated in mood and cognition [148, 149]. 

The finding that antidepressant treatment increases neurotrophic factor expression and hippocampal neurogenesis provided the background and rationale for the neurotrophic theory of depression, as mentioned before. Indeed, both *in vivo* and *in vitro* studies have supported the notion that chronic but not acute antidepressant treatment (fluoxetine, reboxetine, tranylcypromine, clomipramine, imipramine but not desipramine) induces an increase of bromodeoxyuridine (BrdU, a thymidine analog, marker for dividing cells) positive cells number, increasing the proliferation of hippocampal cells and their maturation progress to become adult granule cells. Given that stress induces atrophy and loss of hippocampal cells which contribute to the pathophysiology of depression, and that antidepressant treatment is able to overcome these negative effects by increasing the proliferation and neuronal number, it is possible that such events contribute to the therapeutic actions of antidepressants drugs [60, 150]. Equally, the electroconvulsive therapy and lithium, also increase hippocampal neurogenesis [59, 151]. Therefore, it seems that compounds or other therapies used for depression treatment improve the pathophysiology of depression probably by increasing adult hippocampal neurogenesis. However, it still remains an open question how adult neurogenesis modulates mood and how antidepressants work under this pathway. Concerning this issue, an elegant hypothesis about a functional differentiation along the septo-temporal axis in the hipoccampus has been developed [61]. The authors suggest that neurogenesis in the ventral dentate gyrus may be preferentially involved in the regulation of emotion and the dorsal pole would be modulating learning processes [61]. In such way, the ventral pole would project to mood related areas, such as prefrontal cortex, amygdala, nucleus accumbens. In addition, it is known to possess a very dense serotoninergic innervation, compared with the dorsal part, and a specific topography of mossy cells and hilar interneurons [152]. Furthermore, the exposure to chronic mild stress decreased cell proliferation in the ventral but no in dorsal dentate gyrus, being this effect specifically reversed by the SSRIs escitalopram [153]. These data are promising to understand hippocampus involvement in mood regulation and possibly in the pathophysiology of depression, although the role of BDNF in this putative hippocampal functional division is still unknown.

From available evidence, we could suppose that there is a unique and bidirectional pathway from illness to the recovery that implicates BDNF and hippocampal neurogenesis. However, we are still far away from proving that. In fact, several papers have shown that neurogenesis is not required for the emotional beneficial behavioural effects of the antidepressant fluoxetine in a highly anxious mice strain [154], or of a melanin related compound [155] or even of environmental enrichment [156]. Moreover, several clinical and preclinical studies have suggested that opioids also have a genuine antidepressant effect [157, 158], and therefore it could be possible that they would induce increased neurogenesis as well. However, chronic administration of morphine and heroin opiates, decrease, instead of enhancing, adult hippocampal neurogenesis by about 40% in the granule cell layer [159]. Regarding BDNF hippocampal expression, there is evidence that acute intracerebroventricular administration of opioids increase it [160], but there is no results available about the effect of chronic treatment. Accordingly to these data, it could be suggested that the decrease of new neurons formation might be associated to the negative effects of these drugs of abuse, or that the antidepressant effect of opioids is independent of neurogenesis. While waiting for further studies that may clarify this and other points, we can consider that perhaps a plausible explanation is that there are hippocampal (and neurotrophic) dependent and independent mechanisms involved in the antidepressant effect of classical antidepressant drugs, and that hippocampus neurogenesis is one of the modulator mechanisms that favour mood recovery. In this line, it is still a matter of discussion if the effect of antidepressants on neurogenesis involves always neurotrophins. As a matter of fact, a recent study has shown that noradrenaline activates self-renewing and multipotent neural precursors, including stem cells, by directly acting on beta-3 adrenergic receptors of the subgranular hippocampal zone [161]. Additionally, it is still less clear, at least at the moment, if a reduction of this neurogenic process is underlying human depression. 

#### Lessons from Mutant Animals

As in many other areas in neuroscience, transgenic animals have been used to elucidate the role of endogenous BDNF and TrkB activation on the antidepressant response. In this way, Saarelainen [162] used TrkB.T1 transgenic mice which over express the dominant-negative TrkB.T1 isoform in neurons of the hippocampus and cortex, showing a reduced TrkB signalling in the brain. They have found no differences in immobility behaviour in the forced swimming test between transgenic and wild-type mice, but when imipramine or fluoxetine antidepressant treatment were administrated, they could not observe any significant effect on the immobility behaviour of transgenic animals [162]. This result suggests that the decline of this signalling pathway does not have a main effect on mood models or at least in this model of “behavioural despair” but that inhibition of TrkB signalling leads to a transgenic mouse insensitive to the behavioural effects typical of antidepressant drugs. However, given that TrkB receptor can be activated by both BDNF and NT-3, the next logical step was to explore, in the same paradigm, specific BDNF or NT-3 deficient mice without any pharmacological treatment. To achieve this, heterozygous NT-3^+/-^ and BDNF^+/-^ mice were used, because homozygous mice die early during postnatal development [163, 164]. Thus, NT-3^+/-^, but interestingly not the BDNF^+/- ^mice, showed a normal response to anti-depressants [162]. Additionally, both acute and chronic antidepressant treatment induced TrkB autophosphorylation, and this phosphorylation correlated with the behavioural response of antidepressants [66, 162]. Overall, these findings suggest that antidepressant drugs response takes place through subsequent signalling by TrkB receptor activation in a BDNF-dependent manner. 

In conditional BDNF knockout (KO) mice, in which BDNF has been deleted in broad forebrain regions, Monteggia and collaborators have demonstrated that loss of BDNF in both male and female mice attenuates the actions of the antidepressant desipramine in the forced swimming test [165]. Studies have also examined whether the loss of BDNF signalling, by impairing the function of the TrkB receptor, influences depression-like behaviour. Conditional TrkB KO mice, in which TrkB expression was deleted in the forebrain, resulted in an indistinguishable level of immobility time in the forced swimming test compared with wild-type mice [166]. Moreover, transgenic mice overexpressing the full-length neurotrophin receptor TrkB (TrkB.TK+; isoform responsible for most known effects of TrkB activation) exhibit increased activation of the TrkB/PLCγ pathway, reduced anxiety and depressive-like behaviour and facilitated learning [73, 167]. Taken together, these findings support the notion that forebrain BDNF may be important in mediating antidepressant efficacy and that the increase of BDNF levels is sufficient to produce an antidepressant response. Additionally it appears that activation of TrkB plays a critical role in the behavioural responses to antidepressant drugs, but that the lack of TrkB is not enough to produce depressive-like behaviours. Therefore, as other studies suggest, also the use of transgenic animals indicate that BDNF-mediated TrkB activation is required for the antidepressant behavioural response of antidepressants in behavioural paradigms. In addition, it has been recently proposed that BDNF may have a permissive role in mediating hippocampal dentritic remodelling in CA3 pyramidal neurons, since BDNF(+/-) mice were unable to modify their dentritic structure due to chronic restraint stress [76].

Recently, the protein p11 has received a particular attention by the scientific community researching the mechanisms of depression pathophysiology. The protein p11 is abundantly expressed in hippocampal GABAergic interneurons and it is down-regulated in human and rodent depressive-like states and increased after chronic antidepressant treatment. p11 KO mice are insensitive to the antidepressant actions of BDNF, suggesting this protein is important to mediate the antidepressant-like effects of BDNF [168], and show increased levels of markers for immature neuronal cell survival and neurogenesis [169]. Moreover p11 KO mice have an attenuated response to fluoxetine in measures of neurogenesis and in a neurogenesis-dependent behavioural test [169]. 

Transgenic mice for the serotoninergic system have also been useful in elucidating some of the neurotrophin-mediated mechanisms of depression. Accordingly, it has been recently demonstrated that serotonin transporter (SERT) KO rats, which show a depressive-like behaviour, have reduced BDNF expression in the hippocampus and prefrontal cortex [170]. Additionally, chronic treatment with the antidepressant duloxetine restored the expression of BDNF mRNA-coding exon (IX) in the hippocampus and prefrontal cortex of SERT KO rats through the modulation of selected neurotrophin transcripts, whose expression was up-regulated by duloxetine only in SERT KO rats [171]. The authors thus suggest that the region and isoform-specific increase of BDNF levels may be important to fix normal plasticity that is probably defective in SERT KO mice

Overall these studies in transgenic animals are providing insights about the signalling mechanisms implicated in BDNF-action. Although further studies are still required, the results available until now suggest that BDNF is necessary for a response to antidepressant treatment but that partial or conditional BDNF deletion is not sufficient to produce a depressive-like phenotype.

### Clinical Evidences

#### BDNF in Depressed Patients and Related Post-Mortem Studies

In most clinical studies BDNF levels have been found to be consistently lower in depressed patients than in healthy volunteers [172-179] (review in [180]). These decreases are in some cases correlated with higher scores in specific depression evaluation scales [172]. In *post-mortem* studies, reductions in the expression of pro-BDNF were also seen unilaterally in formalin-fixed paraffin-embedded sections in the hippocampus, but not in dentate gyrus, of MD subjects [181]. There are some exceptions though, with a few studies reporting no differences in plasma or serum BDNF levels between depressed patients and healthy controls [182, 183]. Indeed, serum BDNF levels were lower in bipolar disorder patients during an acute depressive episode than in patients diagnosed for MD [182]. Some studies also suggest a gender specificity in what concerns BDNF levels during depression [173, 175]. Thus, although the application of the Hamilton Depression Rating Scale (HDRS) scores did not detect significant gender differences, both healthy and depressed males showed higher serum BDNF levels than female subjects [175]. 

Biochemical data has also been consistent in indicating a restoration of BDNF levels following antidepressant therapy 177, 178, 184, 185 (review in [180]). Some studies indicate, however, that the changes in BDNF concentrations depend on the antidepressant administered instead of being a general output of antidepressant treatment [186]. The mechanisms contributing to such augment of BDNF upon antidepressant therapy are poorly studied in humans. Nevertheless, the study of Cattaneo and colleagues [184] indicates that BDNF serum increases upon antidepressant treatment are associated with changes in BDNF mRNA levels in leukocytes, suggesting that these cells might play an active role in the mechanisms of action of antidepressant drugs and perhaps may be used as a specific biomarker in the future. Additionally, there is also some evidence that the efficacy of atypical antipsychotics as adjuvant to commonly used antidepressants in refractory depressed patients might involve an increase of plasma BDNF levels [187]. Also, it was found that endurance training induced increased BDNF levels in the hippocampus and an enhanced release of BDNF from the human brain [188]. Indeed, these findings might be related with the beneficial effect that physical exercise seems to have on depressed patients [142-147]. But then again, there is data against this. In fact, BDNF plasma levels were found to be significantly increased during exercise in non medicated patients with moderate depression. This rise, however, was not correlated with improved depression scores, and was of the same magnitude as that observed in healthy subjects, which may be due to the fact that no differences were found in BDNF levels between patients and controls prior to exercise [183]. Sleep deprivation, as adjuvant to antidepressant therapy, seems equally to be associated with increases in BDNF levels [172, 176]. On the other hand, electroconvulsive therapy in depressed patients has been shown to either increase [176, 189], and in remitter patients even restore [176] BDNF levels, or on the contrary to have no effect on BDNF expression [190].

Clinical studies have indicated a relevant association of MD with a significant neuronal atrophy, especially at the hippocampal level [191-193] (more references in the review [194]). This hippocampal degeneration is most likely correlated with the reduced trophic support, as suggested by findings of decreased BDNF and other neurotrophins in *post-mortem* hippocampal tissue of MD patients and suicide victims [181, 195] (review in [196]). Antidepressant therapy, by mechanisms that seem to promote increases in BDNF hippocampal levels and adult neurogenesis, has been shown to block or reverse the loss of hippocampal volume [197] (reviews in [196, 198]), as already discussed. Thus, one question that has aroused from animal and these clinical findings is that the decrease of neurogenesis may be one of the causal factors of depression. Yet, we still do not know whether dentate gyrus function is altered in depressed patients and whether this is restored with the antidepressant treatment. There are just three studies available on depressed patients. Reif and collaborators [199] failed to find changes in proliferation in depression, and antidepressant treatment had no effect. In contrast, Boldrini [200] found a reduction in proliferation in non-treated depression whereas antidepressant-treated patients showed higher proliferation numbers. There is a third report in elderly patients that has found decreased proliferation and reduction in the number of progenitor cells but in agreement with Reif’s study has not found any stimulatory effect upon antidepressant treatment [201]. This discordance may be related to the type of antidepressant medication in combination with the age of these patients. Hence, further studies in preferably larger patient groups are warranted. From a structural point of view, recent data also indicate that even healthy subjects with increased risk for developing depression, either due to familial predisposition or childhood neglect-induced stress, have reduced hippocampal volumes [202-204]. This suggests that lower hippocampal volumes in healthy individuals might indicate a tendency to develop depression [191-192, 202-205] and thus imaging studies may become useful tools to predict a future onset of the disorder. However, it seems unlikely that putative reductions in hippocampal volume are related with neurogenesis because animal studies have shown that volumetric modifications are due to reduced dentritic complexity and not to the impairment of new neurons born [206]. However, although not being a consequence may be a co-existent fact. Another reason that discourages the view of decreased hippocampal neurogenesis or BDNF levels as etiological factors of depression is, as mentioned before, that the blockade of hippocampal neurogenesis [207] or conditional deleted expression of BDNF in male mice [165] do not induce a depressive-like behaviour. Nevertheless, it is still unknown if it is a predisposing factor, that is, if the combination of impaired neurogenesis with genetic predisposition or environmental factors triggers depression appearance. Thus, the role that genetic and environmental factors play together with neurogenenesis and the contribution of each to a future development of the disease is still an issue that needs further investigation. 

#### BDNF Polymorphisms in Depression

The search for variations at the BDNF gene has resulted in the identification of several single nucleotide polymorphisms (SNP) but the rs6265 has been the mostly studied until now. This polymorphism is located at nucleotide position 196 of the human BDNF gene and consists on the substitution of a guanine by an adenine base, which generates the replacement of valine by methionine at codon 66 (Val66Met) in the amino acid sequence. The Val66Met polymorphism has been associated with abnormal intracellular trafficking and regulated secretion of BDNF in cultured hippocampal neurons [208]. Also, there is some piece of evidence suggesting that human subjects having the Met allele in the BDNF gene have abnormal hippocampal activation [208] as well as poorer episodic memory [208] and verbal recognition memory [209]. Other studies of small human cohorts have equally shown that subjects carrying the Met66 allele have smaller hippocampal volumes than Val/Val homozygotes [210-212]. Additionally, reduced grey matter volumes were also observed in the thalamus, parahippocampal gyrus and amygdala of Met66 allele carriers [211]. Conversely, by studying a large cohort of healthy human individuals, others have found that the BDNF Val66Met genotype is unrelated to hippocampal structure or memory performance [213]. Additionally, other studies have equally failed to show a link between the Val66Met polymorphism and memory function [214], and quite on the contrary, have associated carriers of the Met66 allele with better cognitive functioning in the psychomotor and motor domains, suggesting that this allele may confer neuroprotection against the decline of these cognitive functions, at least in patients with systemic lupus erythematosus [215]. In what concerns major depression, Frodl and colleagues [205] have found significantly smaller hippocampal volumes in depressed patients, but also in healthy controls, carrying the Met66 BDNF allele compared with Val/Val homozygotes, concluding that the presence of a Val66Met BDNF polymorphism may predispose human carriers to develop smaller hippocampal volumes and depressive disorders. Reduced grey matter hippocampal volume in Met66 carriers was also associated to elevations in trait depression [216]. However there is some discrepancy among different studies concerning the association between the Val66Met BDNF polymorphism and reduced hippocampal volumes in major depression. As an example, Jessen and partners [191] did observe reduced hippocampal volumes in major depressed patients but an effect of the presence of a Met66 allele on hippocampal volume was not found in either depressed or healthy subjects. Similarly, in depressed elders, the BDNF Val66Met polymorphism was also not significantly associated with smaller hippocampal volumes or impaired cognitive function [217]. Therefore the authors suggest geriatric depression may be mediated through other mechanisms [217]. It is important to note that age, itself, appears to be related to loss of brain volume, poorer execution of memory tasks [213, 218], as well as reduced levels of serum BDNF [218].

Whether the presence of particular BDNF polymorphisms predisposes the individual for having major depression is a question still under investigation (short review in [219]). Indeed, work in rodents support the idea that the Val66Met polymorphism may serve as a genetic predictor of future development of depressive disorders [220-221]. In humans this is corroborated by some studies indicating that Met66 allele carriers are more liable to have geriatric depression than do Val66 allele homozygote individuals [222-224]. Additionally, the BDNF Val66Met polymorphism has been associated with major depression [225], a risk for suicidal behaviour [226, 227] as well as with rumination in healthy adults [228], a behaviour characterized by the tendency to brood and repetitively think about negative information, that is correlated with depression [229]. However, other reports indicate no obvious association of this BDNF variant with the disease itself [230-232], but there seems to be a relation of Val66Met polymorphism to the levels of BDNF. Another matter of discussion is the correlation with the gender of the individual, occurrence of a BDNF polymorphism and risk for developing depression. A recent meta-analysis study revealed that the BDNF Val66Met polymorphism is not significantly associated with depression or with ethnic factors, but seems related with gender, so that the presence of the polymorphism, may predispose the development of major depression in men more than in women [232]. However, a similar gender effect was not found by Ozan and colleagues [175]. In fact it was not detected any association between Met-carriage and mental health status, as evaluated by the Hamilton Depression Rating Scale (HDRS), as well as any gender effect on HDRS scores in the patients. The authors reported, however, that both patients and healthy controls carrying the Val66Met polymorphism exhibited lower BDNF serum levels than Val homozygote subjects, but this was observed regardless of gender [175]. The connection between carrying a BDNF polymorphisms and the individual’s response to antidepressant therapy is also being evaluated by some researchers but to a lesser extent. Indeed, genetic factors are thought to play a key role in both variation of response to treatment and incidence of adverse effects to medication (for review see [233]). Domschke and partners [234] found that the rs7103411, Val66Met (rs6265) and rs7124442 BDNF polymorphisms are related with worse response to antidepressant treatment over 6 weeks in major depression, particularly in the melancholic depression (for rs7103411 and Val66Met) and anxious depression (for rs7124442) clinical subtypes. However other studies report a significantly better therapeutic effect to citalopram [235], milnacipran or fluvoxamine [236] antidepressant drugs in Met66 allele carriers. Moreover, the Val66Met BDNF variant was also found to be associated with better efficacy in the treatment of depressed patients by repetitive transcranial magnetic stimulation (rTMS) [237].

Evidence suggests that BDNF can enhance serotoninergic transmission (review in [238]). In fact, BDNF promotes the survival and differentiation of 5-HT neurons whereas administration of SSRIs increases BDNF expression. Additionally, both BDNF- and serotonin-mediated signalling regulate the development and plasticity of neural circuits involved in mood disorders such as depression and anxiety [238]. It is crucial then to not overlook the genetic impact of carrying serotonin transporter (5-HTT) gene polymorphisms on development of depression, nor the possible interrelation with a BDNF polymorphism. Indeed, evidence suggests that individual genetic variability and the interaction between 5-HTT and BDNF polymorphisms may account for an increased risk to develop depressive-like symptoms. In their case-only design study of ethnically homogenous patients diagnosed with first episode depression, Bukh and partners [239] independently associated the existence of both low activity variants of the 5-HTT-linked polymorphic region in the serotonin transporter gene and the Val66Met BDNF polymorphism with the presence of stressful life events prior to onset of depression. Similarly, the interaction between the 5-HTTLPR serotonin transporter polymorphism and Val66Met BDNF gene variant was significantly associated to depression originated by stressful factors occurring either in childhood [240, 241] or adult life [242].

As a conclusion, there is some clinical evidence for an important effect of the genetic variability in BDNF and serotonin transporter genes on the risk to develop depressive behaviours and depression-associated mood disorders, such as suicide. Additionally, there seems to exist and interaction between these two genes, especially when early childhood and adult stressful events are present. Data also indicate that the Val66Met BDNF polymorphism is associated with alterations in brain anatomy and memory performance, and also that it might play a role on the individual response to antidepressant therapy. There are, however, studies that do not corroborate these findings. The different analytical approaches between the various studies, especially in what respects the size of the sample analyzed, the clinical scales used for diagnosing the existence and/or severity of mood disorder and the discrimination for different racial, gender and age characteristics may account for such discrepancies.

## NEUROTROPHINS AS COMMON PHARMACOLOGICAL TARGETS FOR DEPRESSION AND OTHER NEUROPSYCHIATRIC DISEASES: ALZHEIMER’S, PARKINSON’S, SCHIZOPHRENIA AND BIPOLAR DISORDER

Apart from their role in the pathophysiology of depression, neurotrophins seem to be implicated in other neuropsychiatric diseases as well, suggesting they might be a common target in the mechanisms causing these different neuropathologies. Actually, epidemiological and neurobiological evidences support a strong relationship between depression and dementia and several common pathophysiological mechanisms have been described some of them involving neurotrophin signalling (for review see [243]). In the initial stage of Alzheimer´s disease (AD), for example, cognitive impairment is often accompanied by mood instability and depressive symptoms [244] and the prevalence of AD is higher in persons with a history of major depression [245]. Several data lead to the hypothesis that BDNF deficiency might be one of the bridges between AD and major depression [246]. Indeed, beta-amyloid (Abeta) protein deposits, which are observed in AD patients, seem associated to changes in BDNF content in serum and in cortical regions. Analysis of BDNF content in the serum of 30 patients at two different stages of AD dementia revealed BDNF values are increased in early stages of AD, while they decrease with the course of the disease, correlating with the severity of dementia [247]. The authors speculate that this peculiar pattern of BDNF content changes may represent a compensatory repair mechanism triggered by neurodegeneration in the initial stages leading to increased degradation of Abeta, while at later stages the decreases may account for a deficient trophic support causing Abeta deposition and progressive neurodegeneration [247]. Western blot analysis of *post-mortem* parietal cortex tissue from AD patients has revealed, however, that BDNF levels are reduced already at early stages of disease, which was correlated with loss of cognitive function [248]. These differing findings concerning the levels of BDNF at early stages in AD patients raises the need for more investigation, but studies in transgenic mouse models of AD suggest that the decreased BDNF expression is dependent on the aggregation state of Abeta as well as on large Abeta oligomers [249]. Studies in rodents also show a close association between BDNF expression and Abeta. Thus, intrahippocampal injections of aggregated Abeta(1-42) abolished the increase in serum BDNF levels and induced a significant decrease of BDNF levels in frontal cortex suggesting impaired BDNF regulation during AD [250]. More recently, the administration of Abeta(1-42) induced a decreased NGF and BDNF expression in the prefrontal cortex along with a depressive-like behaviour in rats detected by increases in the forced swimming test immobility [251]. The use of transgenic mouse models of AD indicates that Abeta reduces BDNF signalling by impairing its axonal retrograde transport [252], therefore causing reduced activation of specific receptors. But BDNF is not the only neurotrophin that seems involved in AD. NGF protein levels are also dysregulated in brain tissues from AD patients, and loss of its tyrosine kinase A (TrkA) receptor has also been reported (for review see [253]). As for BDNF, the microtubule-dependent retrograde trafficking of NGF appears to be defective, both in models of age-dependent cholinergic degradation (the aged Fischer 344 rat model, which shows cognitive impairment [254-256]) and transgenic models of Down’s syndrome and AD [257, 258]. In what respects glial cell line-derived neurotrophic factor (GDNF) the sparse available studies also indicate its levels are changed in cerebrospinal fluid and serum of patients with early Alzheimer's disease [259]. Other neurotrophin that seems somehow related both with major depression and AD is tumour growth factor beta 1 (TGF-β1) [243]. Although TGF-β1 is mainly recognized as anti-neuronal survival, their effects occur *via *Trk receptor signalling and possibly by interacting with BDNF as a co-factor [260]. TGF-β1 increases BDNF and Trk expression [260] and reduces NT-3 mRNA levels [261]. Levels of serum TGF-β1 and the allele and genotype frequencies distribution were unchanged in AD patients in comparison to controls [262]. However other investigators found these levels were dependent on the disease progression, being higher in the plasma of AD-mild patients, less higher in AD-moderate patients while no differences were detected between severe AD in comparison to age-matched subjects with no clinical signs of dementia [263]. Also in the TgCRND8a mouse transgenic model of familial AD, TGF-β1 content was up-regulated in cortical brain regions [264]. The low-affinity p75NTR seems also to be an important therapeutic target in order to prevent or ameliorate the symptoms of neuropsychiatric degenerative diseases. Indeed, mice lacking p75NTR show several alterations in central nervous system and cognitive function [265]. Some authors suggest that the increase in the levels of proneurotrophins found in AD, both pro-BDNF and pro-NGF but in particular pro-NGF, may cause p75-mediated neurotoxicity and cellular apoptosis, typically associated to neurodegenerative pathologies. Additionally, the low TrkA to p75 ratio detected in AD patients enhances the activation of p75 by neurotrophins, proneurotrophins as well as Abeta (which has also been found to bind p75), equally contributing to boost neuronal death through p75 signalling (for review see [266]).

Although there is considerably more information available on Alzheimer’s disease, there is also evidence for changes in neurotrophins content occurring in the course of other neurogenerative pathologies such as Parkinson’s disease, schizophrenia and bipolar disorder (for review see [267-270]). By employing a multi analyte profiling proteomics approach in plasma samples from schizophrenic patients, Domenici and colleagues have found that BDNF may be a candidate biological marker for schizophrenia [271]. However there are some contradictory findings concerning BDNF levels in schizophrenia. Thus, increased serum BDNF levels were found in schizophrenic patients that had been chronically medicated with clozapine or other typical and a typical antipsychotics in comparison to healthy controls or to euthymic bipolar disorder patients. The authors suggest that these findings could be related to the course of the disease itself or to the effects of medication [272]. In contrast, other studies reported decreased serum BDNF levels in schizophrenic patients that had also been treated with clozapine or other antipsychotics [273, 274], in accordance with most of the published studies (for references see tables 1 and 2 on [274]). Decreased serum levels of BDNF have also been found in patients with schizophrenia at the onset of the first episode [275] or during a relapse [276], while no changes were detected after 6 months of treatment with typical and atypical antipsychotics when compared to their own levels at the beginning of the study [276]. This implies that the therapeutic strategies followed had no effect on BDNF levels. To add some more controversy to the issue, findings of no changes in plasma BDNF from schizophrenic patients compared to healthy controls have also already been described [38, 277]. There are not so much studies addressing the role of neurotrophins in bipolar disorder but those available report no differences in BDNF levels in serum from patients in euthymia phase compared to healthy controls [272, 278], but decreases were detected during depressive and manic episodes (for review see [267]). Concerning Parkinson´s disease, studies have been addressing the effects of GDNF (or of other ligands of the family, as neurturin) delivery both in experimental animal models of the disease and in clinical trials, in order to amend the disease symptoms and enhance the morphological differentiation and survival of dopaminergic neurons. Nevertheless there are still many steps to go through before reaching major enthusiastic clinical achievements (for review see [269, 270]).

In conclusion, the available data indicate that many neurotrophins represent a common pathogenic factor between depression and other neuropsychiatric disorders. This seems particularly true for BDNF in the case of AD, which co-prevalence with major depression is high. It has indeed been proposed by some authors that the therapeutic use of BDNF itself or of drugs targeting its production may constitute a valid alternative to treat depressed patients with cognitive impairment or AD concomitant with depression [246]. Additionally, with advancing research on the role of neurotrophins, this may reveal valid for other neuropsychiatric diseases as well.

## OTHER NEUROTROPHIC/GROWTH FACTORS IN DEPRESSION

Although not explored to such a great extent as BDNF, the role of other neurotrophins in the pathophysiology of depression is also under investigation. The vascular endothelial growth factor (VEGF) is suggested to have an antidepressant-like effect [46, 279]. Indeed VEGF signalling mediated through its Flk-1 receptor appears to be essential to induce behavioural responses in chronic and subchronic antidepressant models and for antidepressant-induced cell proliferation [46]. Lee and colleagues have also found that transcription of the neuronal VEGF in hippocampal and dentate gyrus neurons is required to evoke antidepressant-like behaviours and that this is mediated by the cAMP cascade [279]. Clinical studies in depressed patients have inconsistently found increased serum levels of VEGF [280], or no changes when comparing control subjects and patients with major depression [281], either before or upon antidepressant medication [282]. In rats, VEGF protein levels were significantly down-regulated in the Flinders Sensitive Line model of depression in the hippocampus and frontal cortex and were unaffected in hypothalamus, corpus striatum, and serum [283]. The basic fibroblast growth factor (FGF2) is known to also have large neurotrophic activity in the adult central nervous system [284, 285]. Some studies also point to be associated with the mechanisms of action of some antidepressant drugs. In fact, antidepressant treatment in rats elicited an increase in FGF2 mRNA and protein in various regions of the brain. The increases were anatomically specific accordingly to the time after injection and to antidepressant drugs administered, but were observed in cortex and hippocampus of the rats [286]. Increased serum levels of FGF2 were equally detected in medication-free depressed patients [280]. Indeed, there seems to exist and interplay between BDNF, VEGF and FGF in what concerns the mechanism of neurogenesis and associated angiogenesis [198].

The role of NGF in depression is also a matter of research. Hellweg and colleagues in his prospective study with depressed patients found no differences in NGF serum levels upon antidepressant therapy [186]. However some data obtained in rodent models of depression indicates that NGF has significant antidepressant effects [287], and that NGF levels are decreased [288], suggesting this neurotrophin might also play a role in the events leading to depression. Also the neurotrophin-3 (NT-3), GDNF and artemin (member of the GDNF family) were found to have decreased mRNA expression in the blood of patients with major depression. Changes were found during the current depressive state, but not in a remissive state, suggesting that these neurotrophins might be involved in the pathophysiology of major depressive disorder and that their mRNA levels are state-dependent [289]. Indeed NT-3 seems to be a potential candidate as antidepressant (for review see [290]) but more studies are still needed. 

In conclusion, data reveals that a wide range of neurotrophic factors besides BDNF may be potential candidates for the development of better and with lesser side-effects treatments for depression. Possibly the interplay between different neurotrophins plays a major role on the pathophysiology of mood disorders, rather than the action of a sole neurotrophin. It remains to establish, though, the contribution of each of these molecules in the events leading to depressive disorder and the possible link between them. More studies focused on these aspects, and not only on BDNF, are therefore needed. 

## NEUROTROPHIN-INDUCIBLE NEUROPEPTIDES IN DEPRESSION: ROLE OF VGF

In the last few years, the notions on the mechanisms of depression have been changing. Neurotrophins appear to be directly associated with this disease but their indirect action, under pathological conditions, on other molecules must also not be forgotten, neither the action that these molecules could exert on neurotrophins. This seems to be the case of VGF (not an acronym), a neuropeptide highly expressed in brain tissue [291-295] that was already demonstrated to be decreased in depressed patients [296]. VGF appears to be the main target of some neurotrophins. Indeed, VGF was primarily identified as a NGF-regulated transcript [297]. Additionally, studies using specific antibodies to neutralize neurotrophins indicated that BDNF is required for VGF expression, at least in some brain regions [298]. Furthermore, the implication of VGF in neurotrophins-synaptic modulation was also suggested by observing that VGF co-localizes with TrkB [299]. However, the roles of VGF in depression barely begin to be studied, being evident the lack of knowledge around this neuropeptide. Until know, data suggest dysregulation of VGF in psychiatric disorders [300]. Recently, Cattaneo [296] observed that depressed patients without treatment showed lower levels of VGF mRNA when compared with controls, which indicates a role for VGF in the pathophysiology of mood disorders. Thakker-Varia and collaborators [301] had also reported decreased levels of this neuropeptide in the hippocampus after the learned helplessness and forced swimming test paradigms of depression. Thus, it became clear that somehow the neurotrophin-inducible neuropeptide VGF has a role and may constitute a target in the treatment of some psychiatric disorders.

There are some studies showing that antidepressants enhancing primarily the levels of serotonin in the synaptic cleft are also able to influence the transcription of synapse-associated proteins and neuropeptides [302-306]. Thakker-Varia and partners [301] showed that VGF is up-regulated not only by BDNF but also by serotonin. They also demonstrated that VGF protein was diminished in the hippocampus of rats submitted to behavioural models of depression [301]. Although serotonin does not regulate all the genes induced by BDNF, the fact that the VGF gene has a CREB binding site [299] suggests an interaction between regulation by serotonin and BDNF. Indeed, after a chronic treatment with imipramine, it was observed an increase in VGF expression and, in the same way, after electroconvulsive therapy, it was observed an increase in VGF mRNA [307-309]. These treatments were also shown to promote the increase of BDNF expression [74, 307, 310]. CREB was shown to be highly activated [311, 312] by phosphorylation of protein kinase A (PKA, [313]) and adenylyl cyclase [314] upon antidepressants chronic administration, leading to increased levels of cAMP after this treatment [315]. In fact, CREB can constitute a post-receptor target that can be influenced by serotonin and noradrenaline in the synaptic cleft [62, 312, 315-318]. Chen and partners [319] verified that over-expression of CREB in two animals models of depression resulted in antidepressant effect. One molecular target of CREB activation is the transcription of BDNF, suggesting their interconnection. Conti and collaborators [320] showed that CREB-deficient mice do not have an altered response to desipramine and fluoxetine when compared to wild type mice, but BDNF up-regulation was abolished. This suggested that during antidepressant treatment, CREB activation is upstream to BDNF. Moreover, it was shown a certain correlation between CREB mRNA activation and BDNF mRNA levels along with its receptor, TrkB, after chronic administration of antidepressants [62, 74]. So, antidepressants promote an increase of the levels of serotonin and/or noradrenaline in the synaptic cleft conducing to the activation and phosphorylation of CREB and, consequently, leading to BDNF gene transcription. Additionally, these actions may be potentiated by the intriguing overlapping circuit observed in some growth factors, mainly BDNF [221, 321], which means that increases of BDNF expression may lead to more BDNF expression. 

VGF seems to be acting downstream of BDNF by having an antidepressant-like effect and enhancing neurogenesis in the hippocampus [301]. Indeed, it is recognized that antidepressants may act promoting neurogenesis and that this event might be mediated through BDNF and activation of the cAMP-CREB pathway [60, 322, 323]. VGF may be involved in this process as indicated by the proliferation of hippocampal progenitors cells, both *in vitro* and *in vivo*, upon chronic VGF treatment [301]. Additionally, the increase in synaptic activity, as observed in hippocampal cultures upon VGF treatment [324], may constitute an alternative mechanism related with VGF antidepressant-like actions. However, it seems likely that actions in neuronal plasticity may explain the short term action of antidepressants while long term action may be due to neurogenesis.

Finally, the influence of exercise must be contemplated because of its well known benefits in improving mood in depressed patients. Recently, Hunsberger and colleagues [325] showed the involvement of exercise in the regulation of the VGF gene and consequently in its action as an antidepressant-like agent. Indeed, exercise and the growth factor pathway have, as principal target, the gene encoding VGF which has been previously shown to influence not only synaptic plasticity [297], as has been discussed so far, as well as metabolism [326]. The administration of a synthetic VGF-derived peptide induced an antidepressant-like response in mice and mutation of VGF in mice induced the contrary [325], suggesting that VGF signalling may constitute a potential therapeutic target for antidepressant drug development.

## CONCLUSION

Depression is a complex disorder that implies multiple neuronal substrates and brain regions. Currently available antidepressants acutely increase monoamine levels but the chronic treatment is required for the onset of antidepressant effect, meaning that enhanced serotoninergic and/or noradrenergic neurotransmission is not the trigger factor for the clinical actions of these drugs. This has lead to the hypothesis that long-term adaptations are necessary for their effects. Among several alterations of intracellular signal transduction pathways and target genes that may contribute, huge attention has received the neurotrophins regulation, especially BDNF. Initially, it was suggested that low levels of BDNF lead to a depression state. However, nowadays, this vision has been modified, highlighting the role of BDNF in the adaptation of neural networks, including neurogenesis, that are responsible for different aspects of mood regulation and antidepressant-like effect. Thus, BDNF expression seems to be dependent of chronic antidepressant treatment. In fact it is plausible that, somehow, neurotrophins may also have functions in the regulation of transmitter release that finally may account for the onset of the antidepressant effect. Thus, the study of neurotrophic factors modulation may provide insight into the modulation of brain function associated with psychiatric disorders, including depression.

## Figures and Tables

**Table 1.** Regulation of Hippocampal BDNF Expression

Factor	Treatment Duration	BDNF Expression	References
**STRESS**			
Immobilization	1, 7 days, (45 minutes/day)	↓	[33]
	45 minutes		[74, 99]
	8 hours		[327]
Unpredictable	10 days, 4-15 weeks	↓	[62, 328, 329, 330-332]
	19 days, 7-11 weeks	=	[333, 91, 83-85, 87]
Footshock	60 minutes, (0,4 mA)	↓	[334]
Social isolation	6 hours	↓	[104]
Social defeat	10 minutes	↓	[335]
	5 weeks	↑	[86]
Maternal deprivation	24 hours, P9	↓	[336]
Swim stress	10 minutes/day, 14 days	↓	[337]
Restraint	4 hours/day, 3 days	↓	[338]
	6 hours/day, 21 days	=	[79]
		↓	[80]
	1 hour/day, 7 days	↑	[82]
**CORTICOSTERONE**	Acute, 7 days	↓	[33, 95, 339-340]
**ADRENALECTOMY**		↑	[339-340]
**ANTIDEPRESSANTS**			
**NRIs**			
Reboxetine	2-14 days	↑	[341]
Desipramine	14, 21 days	↑	[62, 342-343]
	Acute, 14 days	=	[344, 345]
Maprotiline	Acute, 14 days	=	[344, 345]
**SSRIs**			
Fluoxetine	Acute, 14, 21 days	↑	[62, 332, 343, 345-346]
	7 days	=	[320, 342, 344, 346-347]
	4 days	↓	[346]
Paroxetine	Acute, 14 days	↑	[345]
Sertraline	Acute, 14, 21 days	↑	[62, 345]
Citalopram	2, 9 days	↑	[341, 348]
**SNRIs**			
**Dual**			
Venlafaxine	28, 35 days	↑	[83, 349]
**Tricyclics**			
Imipramine	14, 20, 27 days	↑	[332, 338, 350-351]
	28 days	=	[83]
Amitriptyline	21 days	↑	[351, 352]
**Atypical**			
Mianserine	21 days	↑	[74]
	Acute, 14 days	=	[345]
Tianeptine	21 days	=	[79]
**MOR AGONISTS**			
Morphine, DAMGO^1^	Acute	↑	[160, 353]
**MAOI**			
Tranylcypromine	Acute, 2, 14-21 days	↑	[74, 342, 345, 350, 354]
	4, 14, 21 days	=	[344]
**Benzodiazepine**			
Olanzapine	35 days	↑	[349]
**Ketamine**	acute	↑	[123]
	acute, 7, 14 days	=	[114, 91]
**ECT**	10, 21 days	↑	[344]
**Exercise**	1-28 days	↑	[341, 350, 354-358]
**Lithium**	1, 7 days	=	[122]
	14-28 days	↑	[122, 124]

BDNF, brain-derived neurotrophic factor; ECT, electroconvulsive shock treatment; MAOI, monoamine oxidase inhibitor; NRIs, noradrenaline reuptake inhibitors; SSRIs, serotonin selective reuptake inhibitors; SNRIs, serotonin and noradrenaline reuptake inhibitors,

1 [D-Ala2,N-Me-Phe4,Gly5-ol]-enkephalin.

## ABBREVIATIONS

AD: = Alzheimer´s disease
Abeta: = beta-amyloid
BDNF: = Brain-derived Neurotrophic Factor
BrdU: = Bromodeoxyuridine
CREB: = cAMP-related Element Binding Protein
ERK: = Extracellular signal-Regulated Kinases
FGF2: = Basic Fibroblast Growth Factor
GDNF: = Glial Cell Line-derived Neurotrophic Factor
HDRS: = Hamilton Depression Rating Scale
5-HT_2A_: = Serotonin 2A receptors
5-HTT: = Serotonin Transporter
5-HTTLPR: = Serotonin Transporter Promoter Region
IL-1β: = Interleukin-1β
KO: = knockout
mBDNF: = mature BDNF
MAOIs: = Monoamine Oxidase Inhibitors
MAPK: = Mitogen-Activated Protein Kinases
MD: = Major Depression
mGluR2/3: = Metabotropic Glutamate 2/3 Receptors
NGF: = Nerve Growth Factor
NMDA: = N-methyl-D-aspartate receptor
NRIs: = Noradrenaline Reuptake Inhibitors
NT-3: = Neurotrophin-3
NT-4: = Neurotrophin-4
PLCγ: = Phospholipase-Cγ
p75NTR: = p75 Neurotrophin Receptor
rTMS: = Transcranial Magnetic Stimulation
SERT: = Serotonin Transporter
SGZ: = Subgranular Zone of Hippocampus
SNRIs: = Serotonin and Noradrenaline Reuptake Inhibitors
SNP: = Single Nucleotide Polymorphisms
SSRIs: = Selective Serotonin Reuptake Inhibitors
TGF-β1: = Tumour Growth Factor beta 1
Trk: = Tyrosine Kinase Receptors
TrkA: = Tyrosine Kinase Receptor subtype A
TrkB: = Tyrosine Kinase Receptor subtype B
VEGF: = Vascular Endothelial Growth Factor
